# Nurses' Registration Act, 1919

**Published:** 1921-07-16

**Authors:** 


### Nurses' Registration Act, 1919.

### AGREEMENT IN SIGHT.

The Minister of Health received, on July 7, a deputation representing various nurses' organisations. The deputation, which was introduced by Mr. Herbert J. Paterson, C.B.E., M.C., F.R.C.S., Medical Hon. Secretary, Royal British Nurses' Association, consisted of representatives of that body, and of the Matrons' Council, the Registered Nurses' Parliamentary Council, the National Union Trained Nurses, the Fever Nurses' Association, the College of Nursing, and the Professional Union of Trained Nurses. Sir Alfred Mond was accompanied by Mr. L. G. Brock, C.B., an Assistant Secretary of the Ministry.

In introducing the deputation, Mr. Herbert Paterson said that much unrest had been created in the nursing world by the delay in the publication of the Rules under the Nurses' Registration Act for the admission of existing nurses to the Register. They understood that this was due to the difficulty of securing agreement between the English and Scottish Councils as regards the standard of qualification to be required from candidates for admission to the Register. Section 6 of the Act, which dealt with reciprocity, was ambiguous, but it was understood that the law officers of the Crown had advised that the English Council must admit to the corresponding part of the English Register nurses admitted to the general or supplementary parts of the Scottish Register. This being so, it was essential that there should be uniformity of the standard between the two countries, and the English nurses took the strongest objection to the Scottish proposal to admit to the general part of the Scottish Register, and therefore inferentially to admit to the general part of the English Register, Scottish fever nurses who held the certificate of the Scottish Local Government Board and the Scottish Board of Health.

The Minister, in reply, said that it was happily not necessary for him to enter into any discussion on the legal points which Mr. Paterson had raised, since the Secretary for Scotland had now agreed to withdraw the proposal that Scottish fever nurses should be admitted to the general part of the Register. He was entirely in sympathy with the desire expressed by the deputation for uniformity of standard throughout the United Kingdom, and he thought that agreement was now in sight. As far as he was aware the points still outstanding were of relatively minor importance, and he anticipated no difficulty in their adjustment. But as the English Council had not yet received the draft Scottish Rules in their latest form he could not say definitely that all the difficulties had been removed. Sir Alfred Mond added that he had that morning received from the English Nursing Council a print of the Rules submitted for his approval, and he was prepared to sign them at once subject to the reciprocity rule being deferred for the present. If the English Council accepted this suggestion, as he hoped they would, the Register could be opened at once. He was extremely anxious that the Act should be put into operation without further delay, and he undertook to use his influence to secure that uniformity of standard which he believed Parliament had contemplated when Section 6 of the Act was passed.

Mr. Paterson thanked the Minister for receiving the deputation, which then withdrew.

